# Anti-AMPA GluA3 antibodies in Frontotemporal dementia: a new molecular target

**DOI:** 10.1038/s41598-017-06117-y

**Published:** 2017-07-27

**Authors:** B. Borroni, J. Stanic, C. Verpelli, M. Mellone, E. Bonomi, A. Alberici, P. Bernasconi, L. Culotta, E. Zianni, S. Archetti, M. Manes, S. Gazzina, R. Ghidoni, L. Benussi, C. Stuani, M. Di Luca, C. Sala, E. Buratti, A. Padovani, F. Gardoni

**Affiliations:** 1 Neurology Unit, Centre for Neurodegenerative Disorders, Department of Clinical and Experimental Sciences, University of Brescia, Brescia, Italy; 2 Department of Pharmacological and Biomolecular Sciences, University of Milan, Milan, Italy; 3 CNR Institute of Neuroscience and Department of Biotechnology and Translational Medicine, University of Milan, Milan, Italy; 4 IRCCS Carlo Besta, Milan, Italy; 5 III Laboratory of Analyses, Biotechnology Laboratory, Brescia Hospital, Brescia, Italy; 6 Molecular Markers Laboratory, IRCCS Fatebenefratelli S. Giovanni di Dio, Brescia, Italy; 7 International Centre for Genetic Engineering and Biotechnology-ICGEB, Trieste, Italy

## Abstract

Frontotemporal Dementia (FTD) is a neurodegenerative disorder mainly characterised by Tau or TDP43 inclusions. A co-autoimmune aetiology has been hypothesised. In this study, we aimed at defining the pathogenetic role of anti-AMPA GluA3 antibodies in FTD. Serum and cerebrospinal fluid (CSF) anti-GluA3 antibody dosage was carried out and the effect of CSF with and without anti-GluA3 antibodies was tested in rat hippocampal neuronal primary cultures and in differentiated neurons from human induced pluripotent stem cells (hiPSCs). TDP43 and Tau expression in hiPSCs exposed to CSF was assayed. Forty-one out of 175 screened FTD sera were positive for the presence of anti-GluA3 antibodies (23.4%). FTD patients with anti-GluA3 antibodies more often presented presenile onset, behavioural variant FTD with bitemporal atrophy. Incubation of rat hippocampal neuronal primary cultures with CSF with anti-GluA3 antibodies led to a decrease of GluA3 subunit synaptic localization of the AMPA receptor (AMPAR) and loss of dendritic spines. These results were confirmed in differentiated neurons from hiPSCs, with a significant reduction of the GluA3 subunit in the postsynaptic fraction along with increased levels of neuronal Tau. In conclusion, autoimmune mechanism might represent a new potentially treatable target in FTD and might open new lights in the disease underpinnings.

## Introduction

Frontotemporal Dementia (FTD) is a neurodegenerative disorder characterized by behavioural abnormalities, language impairment, and deficits of executive functions^1,2^. FTD is clinically heterogeneous, behavioural variant FTD (bvFTD) and Primary Progressive Aphasias (PPAs) representing the most common phenotypes^1,2^ and being characterized by frontotemporal atrophy, often asymmetric^3^.

In the last ten years, research in FTD field has witnessed a giant step forward in defining the pathogenetic bases of the disease^4^. The identification of mutations responsible of autosomal dominant inherited disorder, namely *Microtuble Associated Protein Tau* (*MAPT*), *Granulin* (*GRN*) and *chromosome 9 open reading frame 72* (*C9orf72*) mutations, has further elucidated the molecular pathways involved in brain depositions of either Tau or TAR DNA-binding protein 43 (TDP43) inclusions^5^. In FTD cases associated with pathogenic mutations in *MAPT*, the Tau accumulation in neurons and glia has been explained in terms of abnormal phosphorylation of the protein, or an altered proportion in the production ratio of the 4R and 3R Tau isoforms^6^; conversely, *GRN* mutation haploinsufficiency and *C9orf72* expansion lead to TDP43 aggregation, with a less defined mechanism^7,8^. However, for the majority of FTD patients without known pathogenic mutations, the molecular pathways related to either Tau or TDP43 protein aggregations are still uncovered. Furthermore, no clinical or neuroimaging data can clearly predict neuropathological trait in FTD patients without pathogenic mutations, and it is unknown whether Tau and TDP43 unfolding represents the primary pathogenic mechanism or whether this might be triggered by external and still undetermined factors.

At present, no risk factors but genetic background have been recognised in FTD. Notwithstanding this absence, an inflammatory contribution to general neurodegenerative disease pathogenesis has long been hypothesized on the basis of large epidemiological studies^9^. In particular, a significantly increased risk of autoimmune disorders represented by inflammatory arthritis, cutaneous disorders, and gastrointestinal conditions has been found in the PPAs along with an elevation of TNF-α levels^10^. Interesting hints have been provided by studies on FTD language variants, in which an autoimmune process was postulated for some cases^11^. Moreover, genome-wide association analysis (GWAS) in FTD found a significant enrichment for elements of immune system involved in antigen presentation, such as HLA-DR5 locus^12^.

Altogether, these findings argue for a potential role exerted by dysregulation of the immune homeostasis in FTD, even though the key-elements are undefined. Recently, we identified anti- α-amino-3-hydroxy-5-methyl-4-isoxazolepropionic acid receptor (AMPAR) antibodies, namely GluA3 subunit autoantibodies, in a few FTD cases screened at our centre^13^. This evidence, along with the recognised central role of AMPARs in regulating Tau processing^14^ and the presence of asymmetric frontotemporal atrophy in autoimmune encephalitis associated with anti-GluA3 antibodies^15,16^, prompted the present study.

In this work, we carried out a screening of GluA3 autoantibodies in a large series of FTD patients and the neurobiological effect of anti-GluA3 autoantibodies was investigated in rat hippocampal neuronal primary cultures and in neurons differentiated from human induced pluripotent stem cells (hiPSCs).

## Methods

### Setting and participants

Patients fulfilling criteria for FTD^1,2^ were consecutively recruited from the Centre for Neurodegenerative Disorders, University of Brescia, Italy. FTD diagnosis was accomplished by extensive neuropsychological assessment and the presence of frontotemporal atrophy at brain MRI scan.

Only patients with available blood sampling and those with no mutations within *GRN*, *MAPT*, or *C9orf72* genes were considered in the present study.

Demographic characteristics were carefully recorded. Diagnosis of bvFTD and PPA was further considered^1,2^. A positive family history suggestive for autosomal dominant disorder was defined in cases with three subjects across two generations with symptoms of dementia, psychiatric illness, or motor neuron disease^17^. The presence of known autoimmune disorder was assessed by a brief interview.

Neuroimaging features were analysed by means of Voxel-Based Morphometry (VBM, SPM12 software package Wellcome Department of Imaging Neuroscience, London; http://www.fil.ion.ucl.ac.uk/spm).

Moreover, 60 healthy controls recruited among spouses and healthy volunteers and aged- and gender-matched with FTD sample, were included in order to set up the cut-off score of anti-GluA3 antibody levels^18^. Twenty-five control subjects who underwent lumbar puncture for headache or chronic neuroapathy and age-matched with FTD sample, were included to test anti-GluA3 antibody levels and cut-off in cerebrospinal fluid (CSF).

For all participants, informed consent in the study was obtained according to sampling protocols that were approved by Ethics Committee of Brescia Hospital, Italy. The study was conducted in accordance with the Helsinki Declaration.

### Serum dosage of anti-GluA3 antibody levels

Serum samples were frozen immediately after centrifugation and stored at −20 °C pending enzyme-linked immunosorbent assay (ELISA). The detection of anti-GluA3 antibodies (peptide A and peptide B) was performed by ELISA according to previously published protocol^18^, with minor modifications.

Peptide synthesized by Biomatik Company (Cambridge, UK), corresponding to portions of the human GluA3 protein (GenBank accession number P42263), were used: (*i*) NNENPMVQQFIQRWVRLDEREFPEAKNAP (GluA3 peptide A, amino acids 274–302) and (*ii*) NEYERFVPFSDQQISNDSASSENRT (GluA3 peptide B, amino acids 399–424). Comparison of the two peptides between human and rat GluA3 indicated a 98% homology.

According to literature data, there is a clear-cut relationship between anti-GluA3 peptide A antibody and anti-GluA3 peptide B antibody dosages^18^; for the purpose of the present study, we measured anti-GluA3 peptide A antibodies in all samples and anti-GluA3 peptide B antibodies in a subset of samples to further prove the association.

Nunc-immuno microtiter 96-well plates (Thermo Scientific, Denmark) were coated with 100 μl of 20 μg/ml of free peptide in phosphate buffered saline (PBS) plus 0.05% Tween 20 overnight at 4 °C. Wells were then treated after blocking buffer (PBS plus 10% bovine serum albumin-BSA), and 100 μl/well of serum (dilution 1:10), tested in duplicate, was added and incubated for 2 hours at 37 °C. After extensive washes, horseradish peroxidase-conjugated rabbit anti-human IgG antibody (Sigma, Germany) was added (dilution 1:5000) and detection was performed with 100 μl of substrate (Tetramethylbenzidine reagent, TMB). Background was measured as optical density (OD) at 450 nm from wells where no serum was added, and subtracted from each measurement.

Inter-assay variability of anti-GluA3 peptide A antibody was less than 15% (correlation analysis = 99.5); Cronbach’s alpha for intra-assay variability was 0.93 (performed on 40 samples).

### CSF analyses

Lumbar puncture was performed in a subset of FTD patients according to a standardized protocol, in the outpatient clinic, at fasting, from 9.00 a.m. to 10.00 a.m, after informed written consent. CSF was collected in sterile polypropylene tubes and gently mixed to avoid gradient effects. Routine chemical measures were determined. CSF total-Tau, phospho-Tau and Abeta 42 concentrations were measured in duplicate by ELISA test (Innotest hTau Antigen kit and Innotest PHOSHO-TAU 181P, Innogenetics, Ghent, Belgium).

The remaining CSF was stored at −80 °C or in liquid nitrogen for subsequent anti-GluA3 antibody dosage. The same protocol used for serum was applied to test anti-GluA3 antibody peptide A, except for plates (Immulon 4HBX 96-well plates, Dynatech, Germany) and the working dilution (1:2 for CSF and 1:4500 for the secondary antibody).

### Confocal imaging

COS7 cells were cultured in Dulbecco’s Modified Eagle’s Medium containing Glutamax (DMEM + Glutamax, Thermo Scientific) supplemented with 10% Fetal Bovine Serum (Euroclone) and penicillin-streptomycin (Thermo Scientific). The day before transfection, COS7 cells were plated in a 12-wells multiwall plate and transfection of rat or human GluA3-GFP (1 μg DNA) was carried out with lipofectamine LTXT^M^ method (Thermo Scientific). 36 hours after transfection COS-7 cells were fixed with 4% Paraformaldehyde (PFA)-4% sucrose in PBS solution for 10 minutes at 4 °C and permeabilized with 0.1% Triton-X-100 in PBS for 15 min at room temperature (RT). After blocking in 5% BSA in PBS for 30 min at RT, cells were incubated with anti-GFP and with anti-GluA3 antibody (Millipore, MAB5416) or with CSF+ or CSF− samples overnight at RT. Cells were then washed in PBS and incubated with secondary antibodies for 1 h at RT. After washes in PBS coverslips were mounted on glass slides with Fluoromount mounting medium (Sigma-Aldrich, St Louis, MO, USA).

Hippocampal neuronal primary cultures were prepared from embryonic day 18 rat hippocampi as described previously^19^. Experiments were performed by using CSF from 6 patients (3 CSF+ and 3 CSF− samples). In particular, we selected 3 CSF+ samples from patients with high concentration of the GluA3 antibody as measured with the Elisa assay (i.e., optical density 450 nm >0.1).

To analyse dendritic spine density, neurons were transfected at 8 days *in vitro* (*DIV8*) with GFP using the calcium–phosphate method. Neurons were incubated at *DIV14* with CSF with anti-GluA3 antibodies (CSF+) or CSF without anti-GluA3 antibodies (CSF−) at 1:20 or 1:100 dilution for 24 h.

Neurons were then fixed for 5 min in 4% paraformaldehyde - 4% sucrose at 4 °C, permeabilized with 0.1% Triton-X-100 in PBS for 15 min at room temperature and immunostained for GFP; primary and secondary antibodies were applied in 5% BSA-PBS. Cells were chosen randomly for quantification from four different coverslips. Fluorescence images were acquired by using the confocal LSM510 Meta system (Zeiss) with a 63x objective and a sequential acquisition setting at 1024 × 1024 pixel resolution; for each image, three to four sections were acquired, and z-projection was obtained. Analysis of dendritic spine density was performed with ImageJ software.

For colocalization studies, hippocampal neurons (*DIV14*) were incubated with CSF+ or CSF− anti-GluA3 antibodies at 1:20 or 1:100 dilution for 24 h, fixed for 5 min in 4% paraformaldehyde - 4% sucrose at 4 °C, permeabilized with 0.1% Triton-X-100 in PBS for 15 min at room temperature and immunostained for GluA3 and PSD-95, a the postsynaptic marker; primary and secondary antibodies were applied in 5% BSA-PBS. Cells were chosen randomly for quantification from four different coverslips.

All fluorescence images were acquired by using the confocal LSM510 Meta system (Zeiss) with a 63x objective and a sequential acquisition setting at 1024 × 1024 pixel resolution; colocalization analysis was performed with the confocal software (Zeiss).

### Preparation and neuronal differentiation of human iPS cells

Human blood sample was collected according to a clinical protocol approved by the local Bioethical Committees of different medical centres. Participating individuals have been informed of the objectives of the study and have signed an informed consent before inclusion in the study. Peripheral blood mononuclear cells (PBMC) were isolated using Ficoll and growth in StemPro®-34 SFM Medium (ThermoFisher), supplemented with L-Glutamine (2 mM, Euroclone), PenStrep (1%, ThermoFisher), SCF (100 ng/mL, ThermoFisher), FLT-3 (100 ng/mL, ThermoFisher), IL-3 (20 ng/mL, ThermoFisher), IL-6 (20 ng/mL, ThermoFisher). To generate iPSC, PBMCs were transduced with 2.0 Sendai virus particles containing four Yamanaka factors using the integration-free CytoTune-iPS Sendai Reprogramming Kit (ThermoFisher). After seven days, transduced cells were plated on matrigel-coated cultures dishes and grown with Essential 8 medium (ThermoFisher). Three to four weeks after transduction iPSC colonies were picked and transferred onto matrigel-coated culture dishes (Corning) for further expansion or analysis. Immunofluorescence and RT-PCR experiments were performed to detect pluripotency markers (Oct 3/4, Lin28, Nanog.and Sox2). Then iPSCs were differentiated to neural precursors (hNPs) via embryoid body method^20,21^. To obtain terminally differentiated neurons, proliferating hNSCs were plated in Neurobasal medium supplemented with B27 w/o vitA (2%, ThermoFisher), PenStrep (1%, ThermoFisher), Glutamax (2 mM, ThermoFisher), NT-3 (10 ng/mL, Miltenyi Biotec), BDNF (10ng/mL, Miltenyi Biotec), GDNF (10 ng/ml, Miltenyi Biotec), Retinoic Acid (1 uM, Sigma-Aldrich) and growth for 50 days. Medium was changed every 2–3 days thereafter.

### Western blot (WB) analysis

WB analysis was performed in total cell homogenate and in Triton insoluble postsynaptic fractions (TIF) purified from hiPSCs differentiated neurons. To purify the TIF, neuronal homogenates were centrifuged at 13,000 g for 15 min at 4 °C. The resulting pellet was resuspended in 150 mM KCl, 0.5% Triton and spun at 100000 g for 1 h at 4 °C. The final pellet (TIF) was homogenized with a glass-glass potter in 20 mM Hepes buffer containing a protease inhibitor cocktail (Complete^TM^).

Protein samples were separated onto an acrylamide/bisacrylamide gel at the appropriate concentration, transferred to a nitrocellulose membrane and immunoblotted with the appropriate primary and HRP-conjugated secondary antibodies. Membrane development was performed with the reagent Clarity Western ECL Substrate (Bio-Rad) or LiteAblot TURBO (Euroclone) and labelling was visualized by Chemidoc Imaging System and ImageLab software (Bio-Rad). For quantification, each protein was normalized against the corresponding tubulin band.

For WB analysis, the following unconjugated primary antibodies were used: polyclonal anti-GluA3 antibody [Synaptic System - #182 203, recognizing the aa861–870 domain of the GluA3 C-tail, thus distant from the two domain (aa274–302 and aa399–402) recognized by the human antibody present in the CSF+ samples]; polyclonal anti-GluA1 antibody (Merck Millipore); monoclonal anti-PSD-95 antibody (NeuroMab); monoclonal anti-tubulin antibody (Sigma-Aldrich).

WB analysis was then performed in differentiated neuron total cell lysate to evaluate Tau and TDP43 expression, and unconjugated primary antibodies were used as follows: polyclonal Tau-5 antibody (Thermofisher) and polyclonal TDP43 antibody (Proteintech); for quantification, each protein was normalized against the corresponding GAPDH band (G9 antibody; Santa Cruz).

### Statistical analysis

Demographic and clinical characteristics between groups were analysed by using Student’s t-test or Chi-Square Test, as appropriate. Spearman’s correlation analysis was carried out. Results were expressed as mean ± standard deviation (SD). Statistical threshold was fixed at p ≤ 0.05; data analyses were performed using SPSS (version 24; SPSS, Chicago, IL).

Statistical evaluations of confocal imaging studies were performed by one-way ANOVA followed by Tukey as post hoc test. When appropriate, experiments were performed in blind conditions. Quantification of WB analysis was performed by means of computer-assisted imaging (Chemidoc) after normalization on tubulin levels. All the group values were expressed as mean ± standard error (SEM). Comparisons between groups were performed by two-tailed unpaired Student’s t-test. Significance levels were defined as *p < 0.05, **p < 0.01, and ***p < 0.001; statistical analyses were performed using the GraphPad Prism statistical package (GraphPad software, La Jolla, CA, USA).

In human differentiated neurons, Tau and TDP43 levels were evaluated after normalization on GAPDH levels by paired Student’s t-test and significance set at *p < 0.05; statistical analyses were performed using SPSS software (version 21, SPSS, Inc., Chicago, IL, USA).

## Results

### Anti-GluA3 antibodies and related features in FTD patients

One hundred seventy-five FTD patients were consecutively recruited, according to inclusion and exclusion criteria (age: 65.7 ± 7.2 years, female 40.6%), and their serum and CSF tested for the presence of anti-GluA3 antibodies and compared to those of healthy controls (mean age: 62.1 ± 11.0 years, female: 63.7%).

Serum anti-GluA3 peptide A antibody dosage was significantly higher in FTD patients (0.41 ± 0.03 OD 450 nm) as compared to healthy controls (0.22 ± 0.14 OD 450 nm) (P < 0.001).

According to literature data^18^, the cut-off score to establish positive anti-GluA3 antibody dosage was calculated evaluating the mean of GluA3 antibody dosage in healthy controls plus 3 standard deviations; thus, the cut-off score was set at 0.64, (=0.22 + 3*0.14). At this established cut-off score, 23.4% of FTD patients (41/175) were positive for serum anti-GluA3 peptide A antibodies. None of the control subjects resulted above the cut-off score.

Serum anti-GluA3 peptide A antibody dosage significantly correlated with serum anti-GluA3 peptide B antibody dosage (n = 47, p < 0.001, Spearman’s rho correlation coefficient = 0.845) and CSF anti-GluA3 peptide A dosage (n = 69, p = 0.001, Spearman’s rho correlation coefficient = 0.446) (see Supplementary Figure 1).

FTD patients with anti-GluA3 antibody had presenile onset (<65 y/o) in 66% of the cases (27/41), 63.4% presented bvFTD (26/41) and 95.2% had no evidence of autosomal dominant inheritance (39/41).

**Figure 1:**
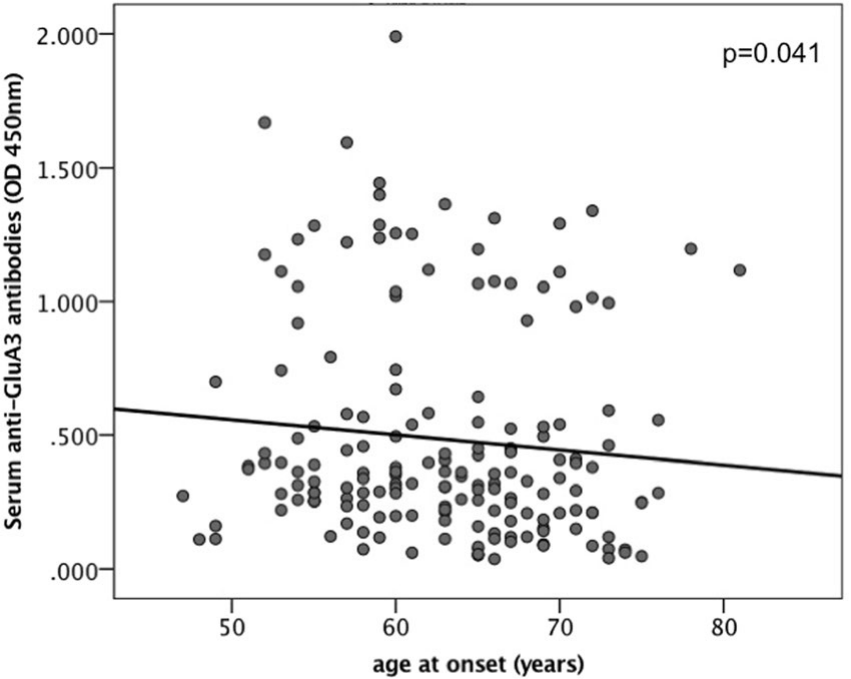
Inverse correlation between age at disease onset and anti-GluA3 antibody titer.

No significant clinical differences between FTD patients with and without anti-GluA3 antibodies were found (see Table 1). However, a significant inverse correlation between anti-GluA3 antibody dosage and age at onset was demonstrated, the higher the serum of anti-GluA3 antibody dosage the lower the age at onset (p = 0.041, Spearman correlation coefficient = −0.155; see Fig. 1). None of FTD patients with anti-GluA3 antibodies presented a positive family history for autoimmune disorders.

**Table 1:** Demographic and clinical characteristics of FTD patients according to serum anti-GluA3 antibody dosage.

Variable	FTD (all)	FTD-GluA3+	FTD-GluA3−	P*
Number	175	41	134	
Age onset, y	62.9 ± 7.0	62.1 ± 7.5	63.2 ± 6.8	*n*.*s*.
Age at diagnosis, y	65.7 ± 7.2	64.8 ± 7.7	65.9 ± 7.1	*n*.*s*.
Sex, F%	40.6 (71)	34.1 (14)	42.5 (57)	*n*.*s*.
FH (AD), %	7.4 (13)	4.8 (2)	8.2 (11)	*n*.*s*.
Education, y	8.9 ± 4.3	9.1 ± 4.0	8.8 ± 4.3	*n*.*s*.
bvFTD, %	70.9 (124)	63.4 (26)	73.1 (98)	*n*.*s*.
CSF Abeta (pg/ml)	839.3 ± 404.5	779.2 ± 339.3	859.9 ± 425.0	*n*.*s*.
CSF Tau (pg/ml)	403.4 ± 278.5	332.4 ± 241.5	426.3 ± 287.3	*n*.*s*.
CSF pTau (pg/ml)	61.2 ± 59.4	48.9 ± 31.4	65.1 ± 65.5	*n*.*s*.

FTD: Frontotemporal dementia; GluA3+: patients with anti-GluA3 antibodies; GluA3−: patients without anti-GluA3 antibodies; y: years; F: female; FH (AD): autosomal dominant family history; bvFTD: behavioural variant FTD; CSF: cerebrospinal fluid. *P-values between GluA3+ vs. GluA3−; n.s.: not significant.

FTD patients with anti-GluA3 antibodies did not differ in CSF Tau and phospho-Tau levels as compared to patients without anti-GluA3 antibodies (see Table 1).

**Figure 2:**
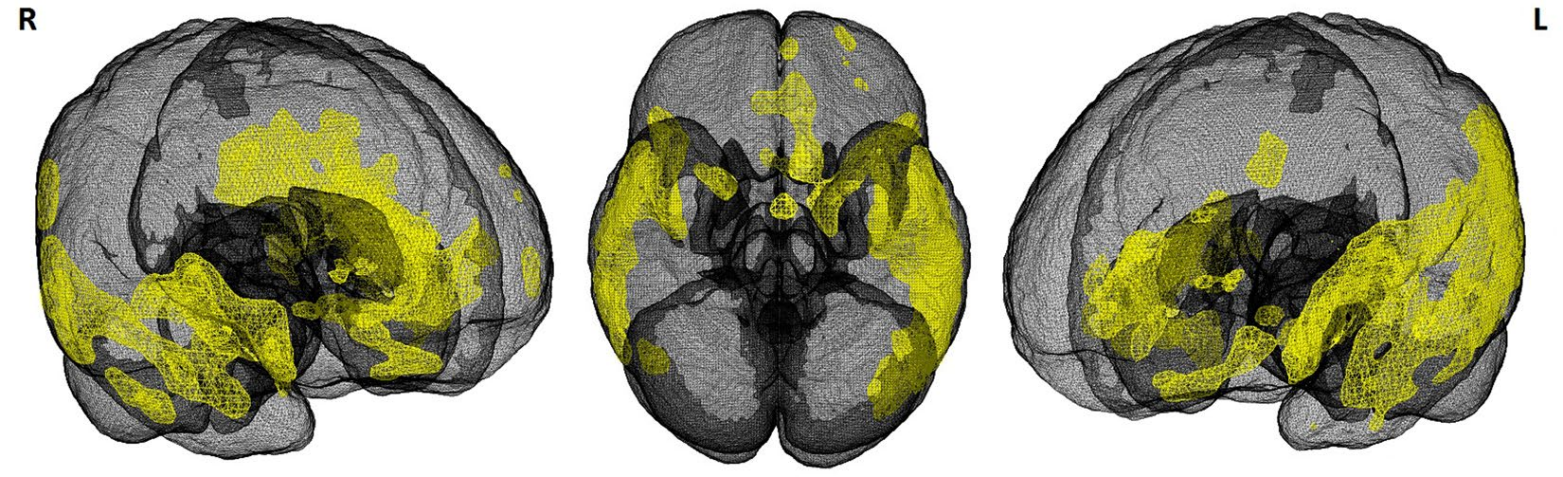
Brain atrophy in FTD patients with GluA3 antibodies. MRI scans were available in 18 FTD patients with anti-GluA3 antibodies (age: 62.4 ± 6.7, gender F: 38.9%) and were compared to 42 healthy controls (age: 61.0 ± 9.1, gender F: 66.7%) (SPM12 software package Wellcome Department of Imaging Neuroscience, London; http://www.fil.ion.ucl.ac.uk/spm). Statistical threshold was set at p < 0.05, Family Wise Error (FWE) corrected, with a minimum cluster size of 50 voxels.

FTD patients with anti-GluA3 antibodies presented bitemporal atrophy at MRI scan (see Fig. 2); no significant neuroimaging differences between FTD patients with and without anti-GluA3 antibodies were found (data not shown).

In regard to CSF GluA3 antibody detection, the cut-off score was set at 0.019 (mean plus 3 standard deviation = 0.004 + 3*0.005), according to values obtained in 25 control subjects (age: 72.2 ± 4.8 years, female 28%). None control subject resulted above the cut-off score.

Out of 69 FTD patients who underwent CSF analysis, 15 resulted above the cut-off scores (21.7%), with significant correlation between serum anti-GluA3 peptide A antibody dosage and CSF anti-GluA3 peptide A dosage (p = 0.001, Spearman’s rho correlation coefficient = 0.446) (see Supplementary Figure 1).

**Figure 3:**
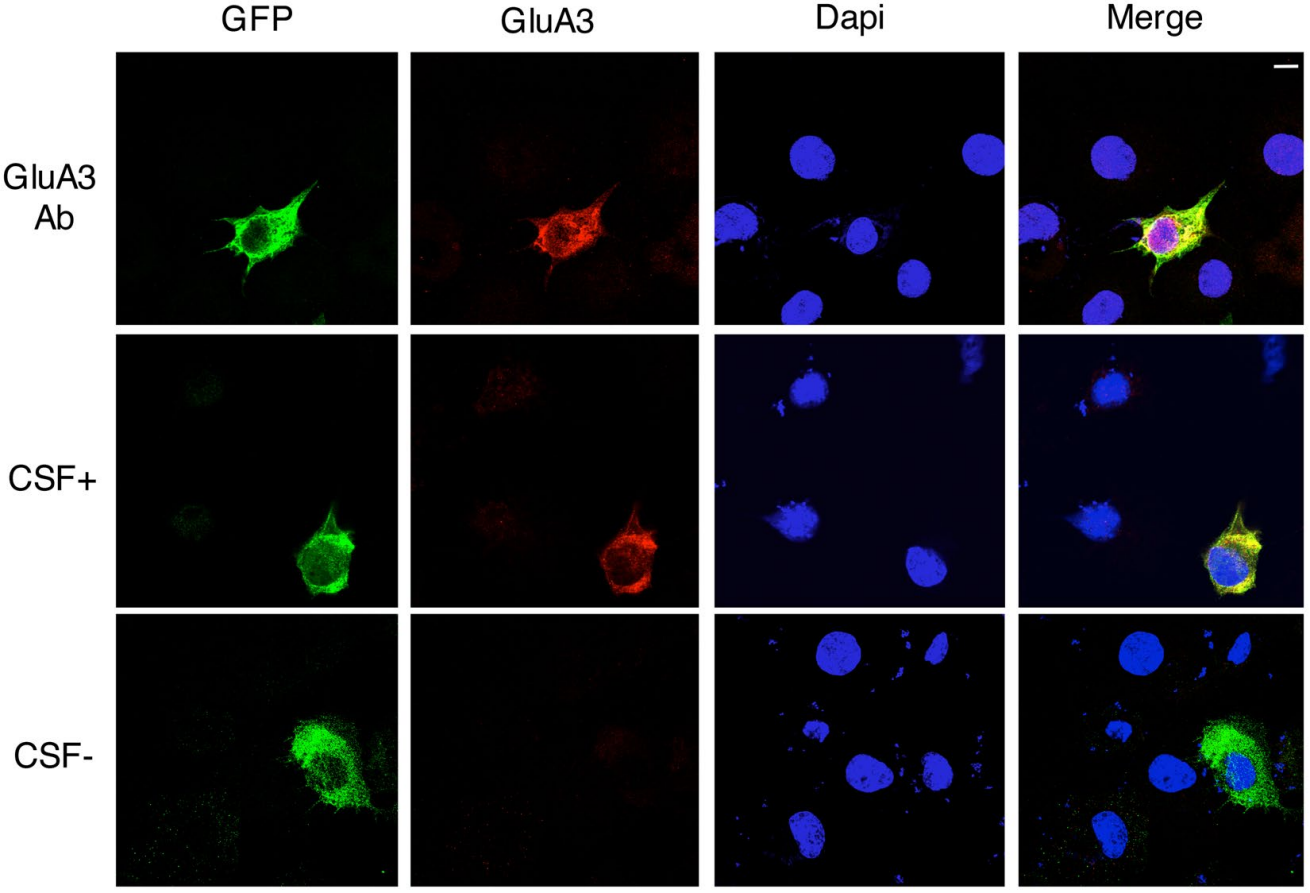
Cell-based assay in heterologous COS7 cell. Confocal imaging of COS7 cells transfected with rat GluA3-GFP (green) and incubated with a GluA3 commercial antibody (upper panels), CSF+ (middle panels) or CSF− (lower panels) for the staining of GluA3 (red). Dapi was used to recognize cell nuclei (blue). Merge panels are shown on the right. Scale bar, 10 μm.

A cell-based assay was used to further confirm the presence of GluA3 antibodies in CSF (see Fig. 3). CSF with (CSF+) or without (CSF−) anti-GluA3 antibodies were used as source of primary antibodies in confocal imaging studies performed in heterologous COS7 cells (not expressing endogenous AMPA receptors) transfected with GluA3-GFP (rat or human sequence). Incubation of COS7 cells with CSF+ lead to a specific staining of cells transfected with rat GluA3-GFP (Fig. 3, middle panel) similar to what has been observed with the use of an anti-GluA3 commercial antibody (Fig. 3, upper panel). Incubation with CSF− did not produce any signal in GFP-GluA3 transfected cells (Fig. 3, lower panel) thus indicating the presence of anti-GluA3 antibodies only in CSF+ samples. The same results were obtained by using the human GluA3-GFP plasmid (Supplementary Figure 2).

### *In vitro* effect of anti-GluA3 antibodies

Two different experimental approaches were used to evaluate possible detrimental effects at neuronal level induced by the presence of GluA3 antibodies in the CSF. In particular, CSF+ or CSF− were added for 24 h to rat primary hippocampal neurons (*DIV14*) and to differentiated neurons from hiPSCs.

#### Rat primary hippocampal neurons

**Figure 4:**
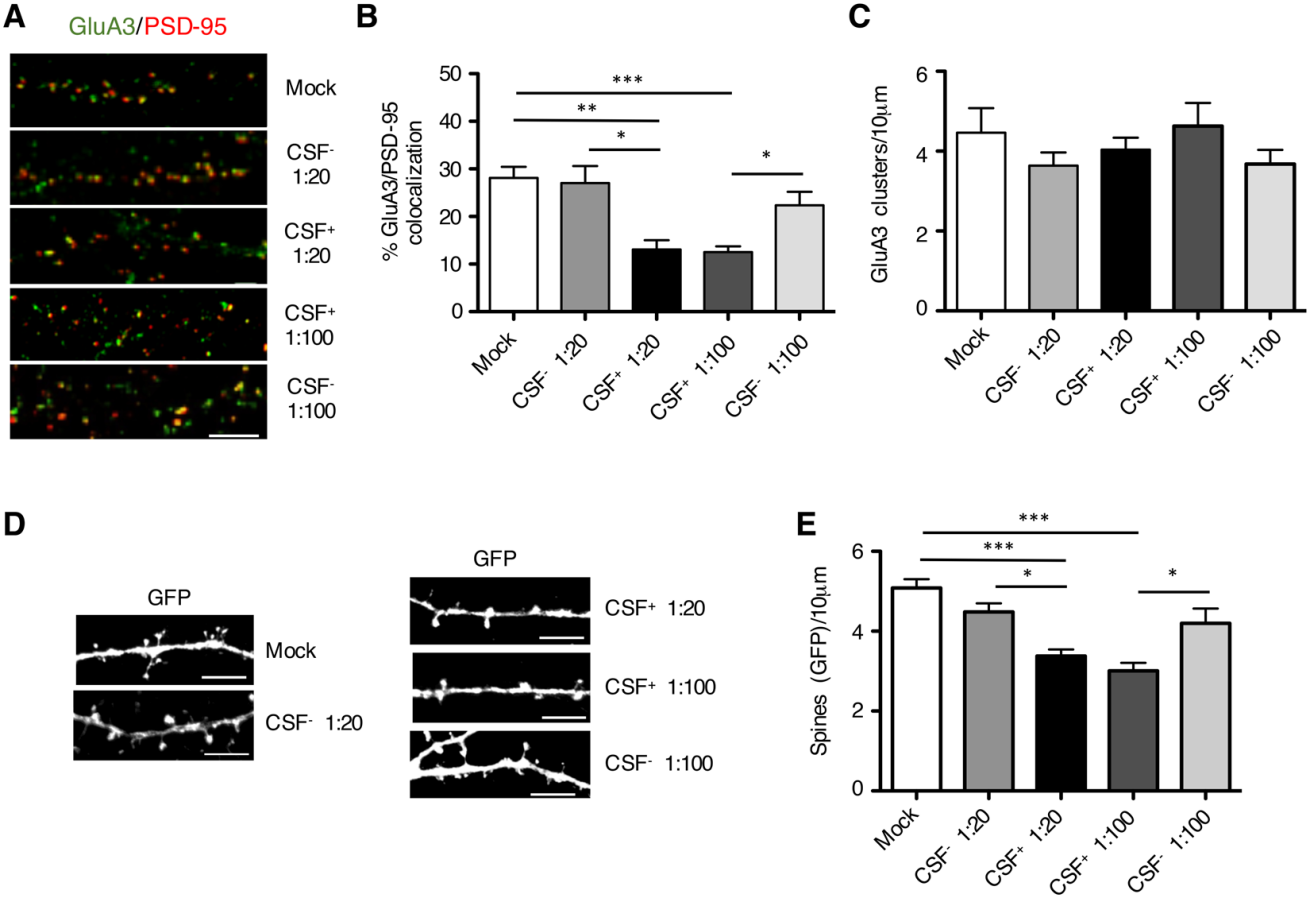
Neurobiological effect of GluA3 antibodies in primary rat neurons. (**A**) Fluorescence immunocytochemistry of GluA3 (green), PSD-95 (red) and MAP2 (blue) in *DIV14* neurons treated with CSF with (CSF+) or without (CSF−) anti-GluA3 antibodies at two different dilutions (1:20 and 1:100) for 24 hours. Scale bar, 5 μm. (**B**) Bar graph representing the percentage of co-localization of GluA3 with PSD-95. Data are presented as mean ± s.e.m, n = 14, one-way ANOVA followed by Tukey as post hoc test; *p < 0.05, **p < 0.01, ***p < 0.001. (**C**) Bar graph representing the density of GluA3 positive cluster along dendrites. Data are presented as mean ± s.e.m., n = 14, one-way ANOVA followed by Tukey as post hoc test. (**D**) Confocal images of rat primary hippocampal neurons transfected at *DIV8* with GFP and immunolabeled at *DIV14* for GFP (green). Scale bar: 5 μm. (**E**), Bar graph showing the quantification of dendritic spine density. Data are presented as mean ± s.e.m., n = 6–8, one-way ANOVA followed by Tukey as post hoc test; *p < 0.05, **p < 0.01, ***p < 0.001.

Treatment of rat primary hippocampal neurons (*DIV14*) with CSF (1:20 and 1:100 dilution) containing anti-GluA3 antibodies (CSF+, Fig. 4A,B) led to a significant decrease of the GluA3 subunit levels at postsynaptic sites, as indicated by the reduction of GluA3 co-localization with the postsynaptic marker PSD-95. Conversely, incubation with CSF lacking the anti-GluA3 antibodies (CSF−, Fig. 4A,B) did not induce any effect on GluA3 co-localization with PSD-95 (***p < 0.001 one-way ANOVA; Tukey post hoc test, mock vs. CSF+ 1:100: ***p < 0.001, mock vs. CSF+ 1:20: **p < 0.01, CSF+ 1:20 vs. CSF− 1:20: *p < 0.05, CSF+ 1:100 vs. CSF− 1:100: *p < 0.05). Interestingly, treatment with CSF+ or CSF− did not induce any modification of GluA3 clusters density thus suggesting that GluA3 antibodies specifically reduce GluA3 postsynaptic levels but not GluA3 total levels along dendrites (Fig. 4A,C).

It is well-known that AMPARs play a key role in the maintenance of dendritic spines at the glutamatergic synapse^22^. Accordingly, we evaluated the effect of the reduction of synaptic GluA3-containing AMPARs on dendritic spine density (Fig. 4D,E). To perform this, rat primary hippocampal neurons were transfected with GFP at *DIV8* and incubated for 24 h with CSF+ or CSF− at *DIV14*. As shown in Fig. 4D,E, the presence of anti-GluA3 antibodies induced a significant reduction of GFP-positive dendritic spines (***p < 0.001 one-way ANOVA; Tukey post hoc test, mock vs. CSF+ 1:100: ***p < 0.001, mock vs. CSF+ 1:20: ***p < 0.001, CSF+ 1:20 vs. CSF− 1:20: *p < 0.05, CSF+ 1:100 vs. CSF− 1:100: *p < 0.05). Again, CSF− did not induce any significant alteration of dendritic spines compared to mock neurons (Fig. 4D,E).

#### - Human iPSCs

**Figure 5:**
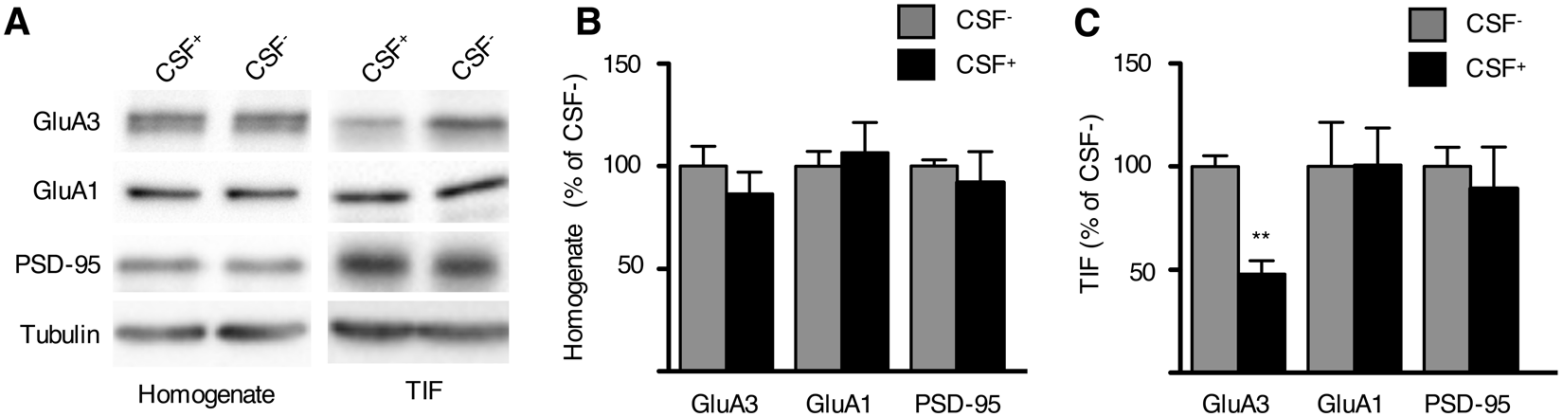
Neurobiological effect of GluA3 antibodies in neurons differentiated from human iPSCs. (**A**) WB analysis from homogenates and triton-insoluble postsynaptic fractions (TIF) of neurons differentiated from human iPSC and incubated for 24 hours with CSF with (CSF+) or without (CSF−) anti-GluA3 antibodies (dilution 1:100). (**B**,**C**) The histogram shows the quantification of the expression levels of GluA3, GluA1 and PSD-95 in homogenate (**B**) and TIF (**C**), normalized on tubulin and expressed as % of CSF−. N = 5 ***p < 0.001, unpaired Student’s t-test.

Next, differentiated neurons obtained from hiPSCs were used to validate and substantiate results obtained in rat primary neuronal cultures. To do this, the hiPSC clones were tested to determine the expression of endogenous pluripotency markers (data not shown). The hiPSC clones were then differentiated into cortical neurons via the generation of neural-rosette intermediate neural progenitors, and they were subsequently differentiated for 80 days. Differentiated neurons were incubated with CSF+ and CSF− (1:100 dilution). After treatment, a biochemical fractionation approach was used to purify a triton-insoluble postsynaptic fraction (TIF)^23^ to be used for WB analysis. As shown in Fig. 5A–C, CSF+ led to a significant reduction of GluA3 levels in the synaptic fraction but not in the total cell homogenate, thus supporting the results obtained by confocal imaging in rat neurons (**p < 0.01, GluA3 TIF CSF+ vs. GluA3 TIF CSF−). Conversely, treatment with CSF+ did not modify synaptic levels of the GluA1 subunit of the AMPARs or the levels of PSD-95, namely the most abundant protein in the excitatory postsynaptic density (Fig. 5A–C).

**Figure 6:**
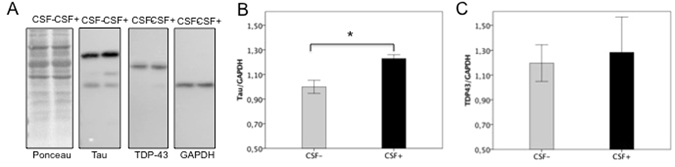
Effect of GluA3 antibodies in neurons differentiated from human iPSc on Tau and TDP43 expression. (**A**) Representative WB analysis from homogenates of neurons differentiated from human iPSc and incubated for 24 hours with CSF with (CSF+) or without (CSF−) anti-GluA3 antibodies (dilution 1:100). (**B**,**C**) The histogram shows the quantification of the expression levels of Tau (**B**) and TDP43 (**C**), normalized on GAPDH; results are expressed as mean±standard errors (SEM), *p < 0.05, paired Student’s t-test.

Finally, we explored the effect of either CSF+ or CSF− incubation on the key-players in FTD, namely Tau and TDP43. As shown in Fig. 6A–C, in the total cell homogenate of differentiated neurons obtained from hiPSCs, treatment with CSF+ lead to an increase of intracellular Tau expression levels as compared to treatment with CSF− (p = 0.017), whilst TDP43 levels were unchanged.

## Discussion

Autoimmunity is a rapidly expanding field of the central nervous system disorders^24^. Autoantibodies to neuronal surface antigens have been described in association with autoimmune encephalopathies, which prominently feature psychiatric symptoms and behavioural disorders^25^. The potential role of these autoantibodies in primary dementia is of increasing interest^13,26,27^.

In the present paper, we reported for the first time the presence of concomitant autoimmune disorder in a significant number of FTD cases, defined by the presence of autoantibodies for the GluA3 subunit of AMPARs both in serum and CSF. In most of the cases, clinical presentations resemble those of bvFTD, with presenile onset, absence of autosomal dominant disorder, and greater bitemporal atrophy. Interestingly, however, antibody titer seem to correlate well with age at disease onset, with earlier symptom onset observed in those patients with higher antibody levels. However, the association between the presence or absence of anti-GluA3 antibodies and related clinical features is weak and needs further investigation. Furthermore, on the present study, we mainly considered serum anti-GluA3 peptide A antibodies, but these well correlated with CSF anti-GluA3 antibodies and serum anti-GluA3 peptide B antibodies, according to literature data^15^.

AMPARs are tetrameric ligand-gated ion channels consisting of the subunits GluA1–4 and play a critical role in mediating fast excitatory neurotransmission. Excitatory glutamatergic synapses mainly express GluA1/GluA2- and GluA2/GluA3-containing AMPARs forming channels that are impermeable to calcium. Notably, the main properties of AMPARs, including kinetics, ligand pharmacology, and membrane insertion/endocytosis are strictly regulated by the subunit composition^28^.

Several evidences have claimed for a possible role of AMPARs in FTD pathogenesis: (*i*) the hyperexcitability of AMPARs contribute to neurodegeneration^29^, (*ii*) in animal model of FTD, the social deficits were accompanied by a change of AMPAR composition^29^; (*iii*) frontal cortex and hiPSCs of bvFTD patients showed changes of AMPARs^30^; and (*iv*) physiological release of Tau protein is mediated by AMPARs^14^.

In this work, we have now demonstrated that anti-GluA3 antibodies are able to modify AMPAR function. We extensively studied the biological role of anti-GluA3 antibodies both in rat hippocampal neuronal primary cultures and in human neurons derived from iPSCs. Overall, our results demonstrated that anti-GluA3 antibodies lead to a reduction of the synaptic levels of GluA3-containing AMPARs both in rat primary neurons and in human neurons differentiated from iPSCs. In addition, the presence of GluA3 antibodies in the CSF is sufficient to induce a significant decrease of dendritic spine density, thus indicating that GluA3 antibodies can trigger not only a molecular event at receptor level but also a more profound morphological alteration of neuronal function.

Our molecular results are in accordance with previous studies performed by using anti-GluA1/GluA2^31^ and anti-NMDA antibodies^32^. Anti-GluA1 and anti-GluA2 antibodies led to a reduction of surface and synaptic of GluA1- and GluA2-containing AMPARs^31^. Similarly, in patients with encephalitis, anti-GluN1 antibodies induced progressive and selective decrease of synaptic NMDAR clusters^32^. However, the effect of anti-AMPAR antibodies on spine density and the capability of these antibodies to act similarly in neurons differentiated from hiPSCs were at present unknown.

Synaptic alterations are strongly correlated with cognitive impairment and memory decline in patients. It is known that AMPARs removal from synaptic sites is critical for this pathway to induce synaptic failure^33^. Notably, blocking AMPARs internalization has been shown to be sufficient to prevent dendritic spine loss. Moreover, in several neurological disorders, AMPAR function and AMPAR-dependent synaptic morphology are altered, thus making these receptors as possible pharmacological targets^34,35^.

At present, we cannot conclusively define whether the presence of anti-AMPA GluA3 receptor antibodies was the consequence of neuronal loss, able to further compromise the on-going neurodegenerative process, or otherwise a trigger event to protein misfolding. However, our data strongly indicate that the presence of these antibodies was detrimental for neurons and AMPAR function.

A further intriguing unanswered question is whether these antibodies are associated with a selective neuropathological hallmark or not. As widely demonstrated by literature, demographic and clinical characteristics are not able to predict neuropathological features in FTD patients. In fact, excluding progranulin dosage, there are no biological biomarkers able to determine the presence of either Tau or TDP43 inclusions^36^. Accordingly, we did not find any significant clinical difference between FTD patients with or without anti-GluA3 antibodies. Neuroimaging data, with a relative symmetric and relative localised predominantly temporal involvement, might suggest Tau-related FTD in first instance^36^. Indeed, it has been demonstrated a direct link between Tau protein and AMPARs: the stimulation of neuronal activity by AMPAR activation induced Tau release from mature cortical neurons in a calcium-dependent way. Tau secretion is therefore a regulatable process, dysregulation of which might contribute to the spread and intracellular accumulation of Tau pathology in disease^14^. The link between Tau and AMPARs was even mediated by Tau protein acetylation, able to impair AMPAR trafficking^37^. In this view, we found increased levels of Tau protein in neurons differentiated from hiPSCs treated with CSF with anti-GluA3 antibodies, and we did not find any significant change in TDP43. However, these represent only preliminary findings and further work on the relationship between AMPAR autoantibodies and autopsy data will be key.

Independently of neuropathological correlations and the still open question whether these antibodies were pathogenic or secondary to preceding neuronal damage, the co-presence of autoimmune process deserves attention. Autoimmune dementias are currently treatable disorders, by different immunosuppressive approaches^24^. In an orphan disorder, as FTD is, immunoglobulin administration and/or long-term rituximab or cyclophosphamine treatment might be claimed in those patients with anti-AMPA antibodies, to reverse or slow the neurodegenerative process.

We acknowledge that the present preliminary study entails several limits. As mentioned before, autopsy correlation analyses should be carried out and the screening for other autoantibodies likely affecting frontotemporal cortex, i.e. anti-NMDA antibodies, should be considered in future studies. A deeper characterisation of Tau processing and Tau protein abnormalities in cases with anti-GluA3 antibodies should also be performed. Furthermore, larger studies are needed to clearly establish the prevalence of FTD cases associated with autoimmune defect homeostasis.

In conclusion, at best of our knowledge, in this work we firstly provided strong evidence for an autoimmune aetiology in a significant proportion of FTD patients. This represents a novel molecular mechanism in FTD, which potentially might benefit of a proper treatment and might open new lights in the underpinnings of the disease.

## Electronic supplementary material

Supplementary Information
