# Publisher Correction: Anti-AMPA GluA3 antibodies in Frontotemporal dementia: a new molecular target

**DOI:** 10.1038/s41598-017-18750-8

**Published:** 2018-01-05

**Authors:** B. Borroni, J. Stanic, C. Verpelli, M. Mellone, E. Bonomi, A. Alberici, P. Bernasconi, L. Culotta, E. Zianni, S. Archetti, M. Manes, S. Gazzina, R. Ghidoni, L. Benussi, C. Stuani, M. Di Luca, C. Sala, E. Buratti, A. Padovani, F. Gardoni

**Affiliations:** 1 Neurology Unit, Centre for Neurodegenerative Disorders, Department of Clinical and Experimental Sciences, University of Brescia, Brescia, Italy; 2 Department of Pharmacological and Biomolecular Sciences, University of Milan, Milan, Italy; 3 CNR Institute of Neuroscience and Department of Biotechnology and Translational Medicine, University of Milan, Milan, Italy; 4 IRCCS Carlo Besta, Milan, Italy; 5 III Laboratory of Analyses, Biotechnology Laboratory, Brescia Hospital, Brescia, Italy; 6 Molecular Markers Laboratory, IRCCS Fatebenefratelli S. Giovanni di Dio, Brescia, Italy; 7 International Centre for Genetic Engineering and Biotechnology-ICGEB, Trieste, Italy

Correction to: *Scientific Reports* 10.1038/s41598-017-06117-y, published online 27 July 2017

The PDF version of this Article was inadvertently replaced after publication. This has now been corrected; the HTML version of the paper was correct from the time of publication.

