# Supplementary material for: Anti-AMPA GluA3 antibodies in Frontotemporal dementia: a new molecular target
Source: Sci Rep. 2017 Jul 27;7:6723. doi: 10.1038/s41598-017-06117-y (PMC5532270; doi:10.1038/s41598-017-06117-y)
Supplement: Supplementary file 1 — Supplementary Information [file 41598_2017_6117_MOESM1_ESM.pdf]

Anti-AMPA GluA3 antibodies in Frontotemporal dementia: a new molecular target

Borroni B*, Stanic J, Verpelli C, Mellone M, Bonomi E, Alberici A, Bernasconi P, Culotta L, Zianni E, Archetti S, Manes M, Gazzina S, Ghidoni R, Benussi L, Stuani C, Di Luca M, Sala C, Buratti E, Padovani A, Gardoni F*

Suplementary Figure 1

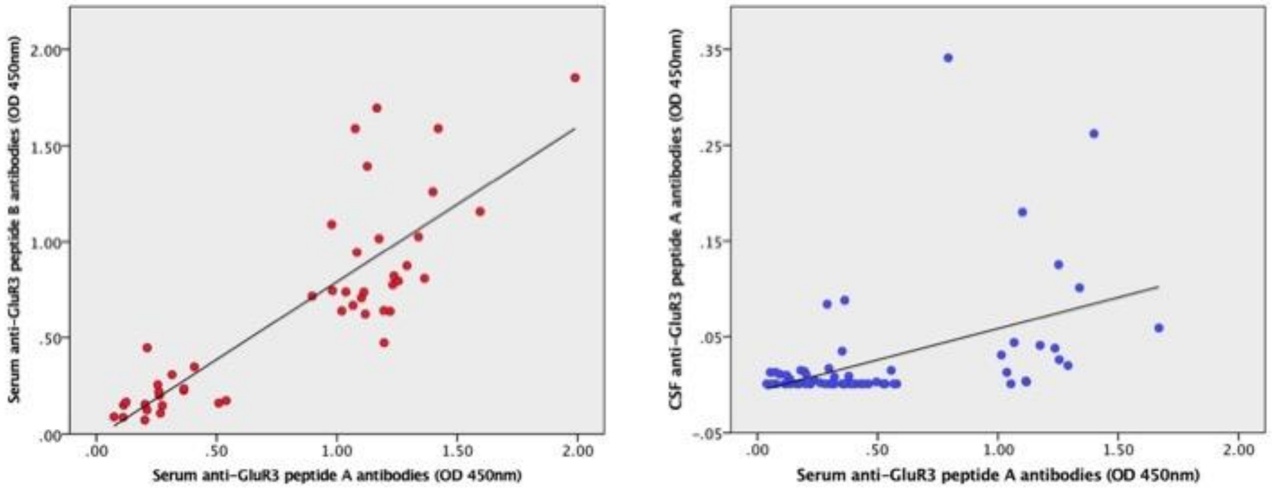

Legend to Supplementary Figure 1. Correlation between serum anti-GluA3 peptide A antibody dosage and serum anti-GluA3 peptide B antibody dosage (panel A) and between serum anti-GluA3 peptide A antibody dosage and CSF anti-GluA3 peptide A dosage (panel B).

See text for details.

Supplementary Figure 2

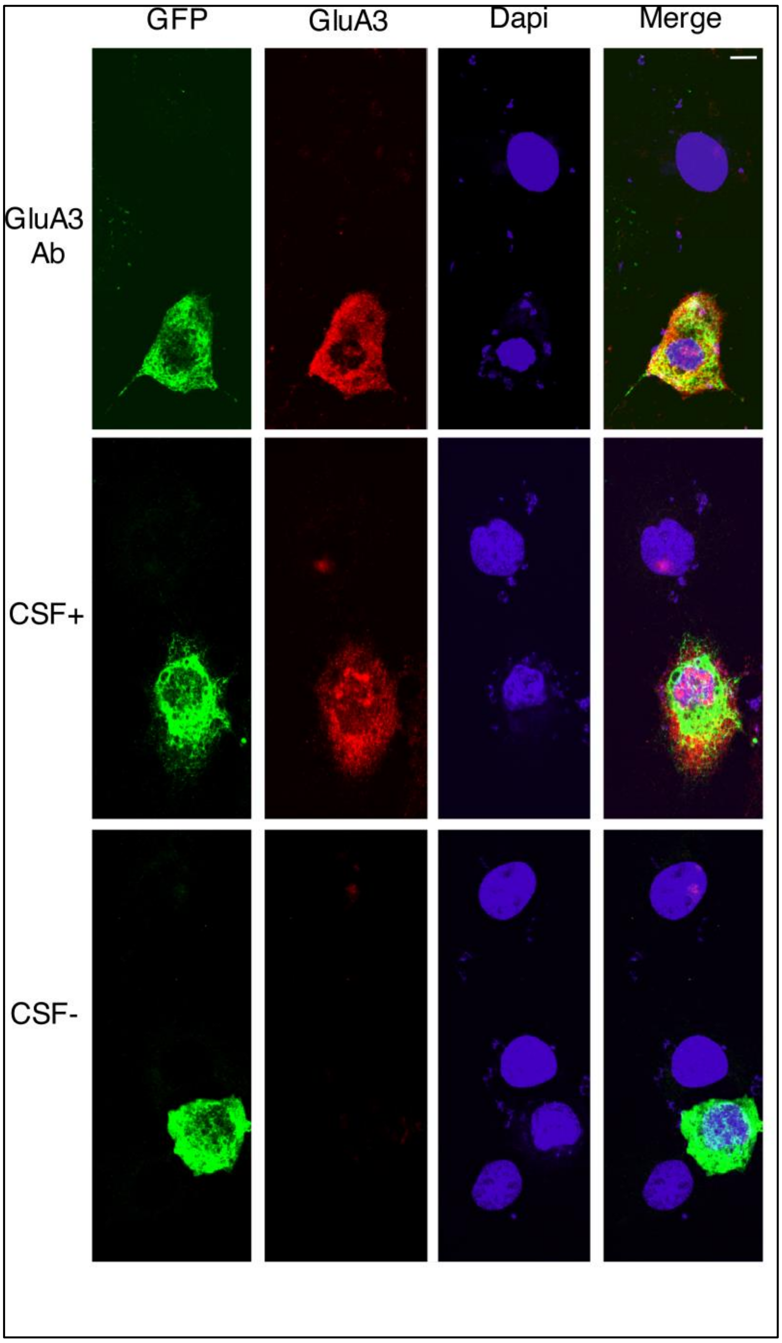

Legend to Supplementary Figure 2. Cell-based assay in heterologous COS7 cell.

Confocal imaging of COS7 cells transfected with human GluA3-GFP (green) and incubated with a GluA3 commercial antibody (upper panels), CSF+ (middle panels) or CSF- (lower panels) for the staining of GluA3 (red). Dapi was used to recognize cell nuclei (blue). Merge panels are shown on the right. Scale bar, 10 μm.
